# Proteomics of Stored Red Blood Cell Membrane and Storage-Induced Microvesicles Reveals the Association of Flotillin-2 With Band 3 Complexes

**DOI:** 10.3389/fphys.2018.00421

**Published:** 2018-05-04

**Authors:** Michel Prudent, Julien Delobel, Aurélie Hübner, Corinne Benay, Niels Lion, Jean-Daniel Tissot

**Affiliations:** ^1^Laboratoire de Recherche sur les Produits Sanguins, Recherche et Développement Produits, Transfusion Interrégionale CRS, Épalinges, Switzerland; ^2^Faculté de Biologie et de Médecine, Université de Lausanne, Lausanne, Switzerland

**Keywords:** band 3, flotillin-2, immunoprecipitation, proteomics, red blood cells, storage, transfusion

## Abstract

The storage of erythrocyte concentrates (ECs) induces lesions that notably affect metabolism, protein activity, deformability of red blood cells (RBCs), as well as the release of oxygen. Band 3 is one of the proteins affected during the *ex vivo* aging of RBCs. This membrane protein is an anion transporter, an anchor site for the cytoskeleton and other membrane proteins as well as a binding site for glycolytic enzymes and bears blood group antigens. In the present study, band 3 complexes were isolated from RBCs stored for 7 and 42 days in average (*n* = 3), as well as from microvesicles (*n* = 3). After extraction of membrane proteins with a deoxycholate containing buffer, band 3 complexes were co-immunoprecipitated on magnetic beads coated with two anti-band 3 antibodies. Both total membrane protein extracts and eluates (containing band 3 complexes) were separated on SDS-PAGE and analyzed by bottom-up proteomics. It revealed that three proteins were present or absent in band 3 complexes stemming from long-stored or short-stored ECs, respectively, whereas the membrane protein contents remained equivalent. These potential markers for storage-induced RBC aging are adenylosuccinate lyase (ADSL), α-adducin and flotillin-2, and were further analyzed using western blots. ADSL abundance tended to increase during storage in both total membrane protein and band 3 complexes, whereas α-adducin mainly tended to stay onto the membrane extract. Interestingly, flotillin-2 was equivalently present in total membrane proteins whereas it clearly co-immunoprecipitated with band 3 complexes during storage (1.6-fold-change, *p* = 0.0024). Moreover, flotillin-2 was enriched (almost threefold) in RBCs compared to microvesicles (MVs) (*p* < 0.001) and the amount found in MVs was associated to band 3 complexes. Different types of band 3 complexes are known to exist in RBCs and further studies will be required to better understand involvement of this protein in microvesiculation during the storage of RBCs.

## Introduction

The organization of the RBC membrane is tightly associated to band 3 macrocomplexes and cytoskeleton. Band 3 is the major protein in RBC membrane and is reported as three major complexes. The first one is the band 3 dimer-ankyrin complex attached to the spectrin chains, the second one is the junctional complex where the band 3 (as dimer) is attached to the cytoskeleton via a complex of adducins, protein 4.1 and actin and the last one is a free band 3 dimer (Kodippili et al., 2009; Burton and Bruce, 2011; Mankelow et al., 2012; Lux, 2016). The knowledge on the composition and organization of the multi-protein complexes in RBC membrane has evolved during the last decades, from the membrane skeleton (Byers and Branton, 1985) to the binding of Rh proteins (Bruce et al., 2003), the role of protein 4.1 (Salomao et al., 2008), protein 4.2 (Satchwell et al., 2009), adducins (Anong et al., 2009), dematin involved in actin binding (Khan et al., 2008) and in junctional complex integrity (Lu et al., 2016), and ankyrin in band 3 tetramer stability (Satchwell et al., 2016). The multiple role of these complexes encompasses the cell deformability required to fulfill the RBC function of O_2_ delivery to tissue and organs (Mohandas and Gallagher, 2008; Kodippili et al., 2009; Burton and Bruce, 2011), in gas exchange process (Bruce et al., 2003) and in metabolism regulation (Chu et al., 2008).

These functions are altered during the storage of RBCs under blood banking conditions (i.e., stored at 4°C up to 42 or 56 days depending on the various additive solutions used). *In vitro* studies have shown an increase in hemolysis (depending on additive solution and manufacturing) and a leakage of potassium, an increase in volume and cell rigidity (Hess et al., 2000; Gov and Safran, 2005; Flatt et al., 2014; Sparrow et al., 2014), the cell shape changes (Blasi et al., 2012; Bardyn et al., 2017b; Roussel et al., 2017), an accumulation of MVs (Rubin et al., 2008; Sparrow et al., 2014), metabolic and antioxidant modulations (ATP and 2,3-DPG depletion), and pH lowering (Hess et al., 2000; Sparrow et al., 2014; Bordbar et al., 2016; Bardyn et al., 2017a), what is called storage lesions (Tinmouth and Chin-Yee, 2001; Hess and Greenwalt, 2002; D’Alessandro et al., 2015; Prudent et al., 2015b; Bardyn et al., 2017b). At the protein level, these storage lesions induce the aggregation and degradation of band 3, leading to the formation of neoantigens recognized by the macrophages for the cell clearance (Bosman et al., 2008), accumulation of hemoglobin, antioxidant and metabolic enzymes at the membrane such as peroxiredoxin-2 (Antonelou et al., 2010; Rinalducci et al., 2011), degradation of proteins and decrease in spectrins and ankyrin contents (D’Amici et al., 2007; Bosman et al., 2008), accumulation of oxidized proteins (in particular at the cytoskeleton) (Antonelou et al., 2010; Delobel et al., 2012; Delobel et al., 2016).

Vesiculation is another mechanism related to the aging of RBCs. *In vitro*, it serves to remove damaged materials [such as oxidized proteins (Delobel et al., 2012)] and senescence molecules (phospatidylserine exposed at the RBC surface, IgG, neoantigen on band 3), which postpones the cell removal and increases their survival upon transfusion (Bosman et al., 2008; Willekens et al., 2008). The number of MVs increases exponentially during storage (Rubin et al., 2008) and it has been shown that after an incubation of 1 h at 37°C, long-stored RBCs exposed even more phosphatidylserine, release more MVs and potassium (Burger et al., 2013), which could impact transfusion efficiency (Cognasse et al., 2015; Said et al., 2017). Moreover, MVs promote the generation of thrombin (Rubin et al., 2013), which may be viewed as representing a potential hemostatic agent [as shown by reduced bleeding time and blood loss in thrombocytopenic rabbits and in Plavix^®^-treated rats (Jy et al., 2013)], or maybe promoters of thrombotic events (Tissot et al., 2013). MVs are formed at cytoskeleton-free spaces within the membrane and microvesiculation is expected to be a lipid-raft-based process affected by the loss of ATP (Sens and Gov, 2007; Gov et al., 2009). Lipid-raft (Ciana et al., 2014) proteins like stomatin and flotillins were found to decrease in RBC membranes during storage (Kriebardis et al., 2007), and stomatin to be enriched in MVs (Salzer et al., 2002, 2008).

Band 3 complexes play a key role in membrane organization and could be affected by the storage. In the present article, band 3 complexes were isolated from RBCs stemming from short and long-stored ECs as well as storage-induced MVs, in order to better decipher the fate of band 3 complexes during storage.

## Materials and Methods

### Chemicals

Acetonitrile, deoxycholic acid (DC), NaCl and ponceau S were from Sigma (Sigma-Aldrich, Steinheim, Germany), and bromophenol blue, Coomassie Bue Brilliant R-250 and FA from Fluka (Fluka Chemie, Buchs, Switzerland). Tween-20 was bought from Roche Diagnostics (Mannheim, Germany). DTE, glycerol and urea were purchased from MP Biomedicals (Illkirch, France), ammonium bicarbonate and iodoacetamide from ICN Biomedicals (Aurora, Ohio, United States), 0.9% NaCl from Baxter (Volketswil, Switzerland), 10x PBS (1x PBS eq. to 137 mM NaCl, 2.7 mM KCl, 8.1 mM Na_2_HPO_4_ and 1.8 mM KH_2_PO_4_) from Laboratorium Dr. G. Bichsel (Interlaken, Switzerland), EDTA from Merck (MSD Merck Sharp & Dohme, Luzern, Switzerland), Tris-HCl from BIO-RAD (Hercules, CA, United States), ethanol from Thommen-Furler AG (Rüti bei Büren, Switzerland), Top Block from Lubio Science (Luzern, Switzerland), benchMark Protein Ladder (prestained or not) from Invitrogen (Carlsbad, CA, United States), FITC mouse anti-Human CD47 antibody from BD Pharmingen (BD Biosciences, Franklin Lakes, NJ, United States), and Sequencing Grade Modified Trypsin (Trypsin) from Promega AG (Dübendorf, Switzerland). Deionized water (18.2 MΩ⋅cm) was prepared using a Purelab option Q-15 (Elga LabWater).

### Blood Samples

Erythrocyte concentrates were prepared from whole blood donations. Briefly, 450 ± 50 mL of blood from healthy donors were mixed with 63 mL of citrate phosphate dextrose anticoagulant solution and left at 22°C overnight (NGR6428B, Fenwal, Lake Zurich, IL, United States). All blood components (i.e., RBCs, plasma and white blood cells- and platelets-containing buffy coat) were separated upon centrifugation at 3,500 *g* for 14 min. The separated components were then distributed among the sterile inter-connected blood bags by applying a semi-automated pressure (on an Optipress II, Fenwal, United States) on the centrifuged original blood donation bag. The RBCs were then transferred into a SAGM-containing bag to a total volume of 275 ± 75 mL and a hematocrit of 0.6 ± 0.1 *v/v*. A leukodepletion step was performed by gravity-based filtration. ECs were finally stored at 4°C. ECs that did not meet the quality criteria for blood transfusion, e.g., low hemoglobin content or a slightly too small volume, were used under the signed assent of blood donors.

No research on genetic material was carried out. Therefore, no specific ethical processing was required and these samples were used in agreement with the local legislation (Loi fédérale relative à la recherche sur l’être humain, LRH – RS 810.30” and “the “Ordonnance relative à la recherche sur l’être humain, ORH – RS 810.301”).

### Preparation of RBC Membranes

Four times 6 mL of ECs were collected from: three ECs (bags A, B, and C) after 5 days, 7 days, 6 days, and 41 days, 43 days, 42 days, respectively; and three others (bags D, E, and F) after 50, 38, and 42 days, respectively, for experiments on MVs. RBCs were washed twice in 0.9% NaCl (2 v) and spun down at 2,000 *g* during 10 min at 4°C. RBCs were lyzed by incubation 1 h at 4°C in a hypotonic 0.1x PBS solution under agitation (2 v of 0.1x PBS for 1 v of packed cells).

Membranes from bags A to F were separated by ultracentrifugation at 100,000 *g*, 30 min, 4°C, and washed twice with 0.9% NaCl before transferring to 1.5-mL tubes and washed 4 to 8 more times (centrifugation at 21,500 *g*, 30 min, 4°C) until to obtain pellets as white as possible. RBC membranes were stored at -70°C until protein extraction.

Microvesicles were harvested from the whole bags D, E, and F (approximately 4 to 6 times 40 mL plus one tube of 6 mL) after centrifugation at 2,000 *g* during 10 min at 4°C (the 6-mL tubes of RBCs were processed as before for membrane isolation). Cell-free supernatants were collected and ultracentrifuged at 100,000 *g*, 1 h, 4°C. MVs-containing pellets were washed two times with NaCl 0.9%. Then, the pellets were suspended in 1 mL of NaCl 0.9% and transferred to 1.5-mL tubes and stored at -28°C. MVs were quantified by flow cytometry as previously described by labeling 5 μL of samples with 5 μL of FITC mouse anti-human CD47 (Delobel et al., 2012).

### Protein Extraction

Total membrane proteins were extracted fresh daily under native conditions from pelleted membranes with DC buffer: 1% DC in 50 mM Tris-HCl, 150 mM NaCl, pH 8.1. A final centrifugation was performed at 21,500 *g*, 30 min, 4°C. The supernatant, containing membrane proteins and cytoskeleton (Delobel et al., 2012), was saved at 4°C. Protein amounts were determined using the Bradford Protein Assay (BIO-RAD, United States), with a calibration curve composed of BSA dilutions.

Proteins from 80 million MVs were extracted with 400 μL of DC buffer (25 million for bag E) and were quantified using a nanodrop 2000c (Thermo scientific).

### Isolation of Band 3 Complexes

Band 3 complexes were captured using Co-IP based on magnetic beads (Dynabeads M-270 Epoxy, Invitrogen, Oslo, Norway). Antibodies that target internal (extracellular) and N-terminal (intracellular) parts of band 3 sequence (BRIC 6 and BRIC 170, respectively, from NHS Blood and Transplant, Bristol, United Kingdom) were covalently bound to the beads using a standard protocol from the supplier (105 μg of antibodies in total on 7.5 mg of beads). For the experiments with the bags A to F, 2×6 sets of beads (containing 7.5 mg of beads each) were prepared, pooled and split in 2×6 equivalent tubes for homogeneity. Antibodies-coated beads were stored at 4°C before Co-IP.

**FIGURE 1:**
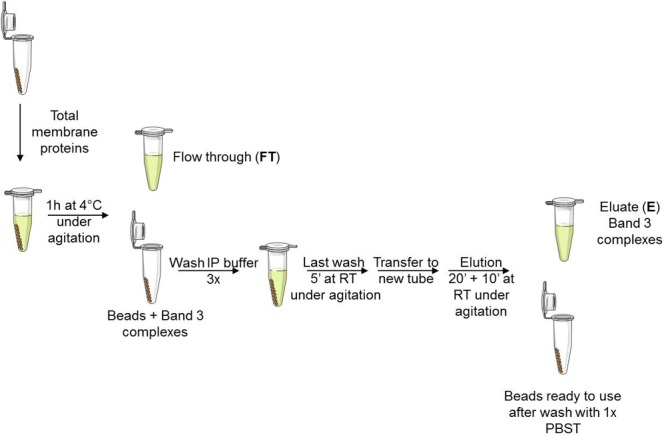
Workflow for Co-IP of band 3 complexes. Magnetic beads were covalently coated with anti-band 3 antibodies (BRIC 6 and 170 specific to internal and N-ter band 3 sequence, respectively). Membrane proteins were extracted with 1% DC buffer. Beads were previously equilibrated with 1x IP buffer. Wash buffer: 1x IP buffer, Last wash buffer: 0.5 M NH_4_OAc, 0.5 mM MgCl_2_, 0.1% Tween-20. Elution buffer: 0.5 M NH_4_OH, 0.5 mM EDTA. Beads were finally washed with 1x PBST and saved at 4°C for further co-IPs.

Co-IPs were performed as shown in **Figure 1**. Beads were washed with 1x IP buffer (provided by the supplier). The tube was placed on a magnet which allows the beads to collect at the tube wall and remove the supernatant (to be saved or not). Two hundred μL of total membrane protein or MV extracts, that contained approximately 400 μg of proteins, were added on the beads and incubated 1 h at 4°C under agitation. The proteins that were not immunoprecipitated were collected in the FT and the beads containing the band 3 complexes were carefully washed 3× with 1× IP buffer followed by a final 5 min wash at RT under agitation in the last wash buffer (LWB, 0.5 M NH_4_OAc, 0.5 mM MgCl_2_, 0.1% Tween-20). Finally, the bead suspension was transferred to a clean tube for elution of band 3 complexes with 500 μL of elution buffer (EB, 0.5 M NH_4_OH, 0.5 mM EDTA) by incubating 20 min at RT under agitation followed by a second elution with 500 μL of EB 10 min. Eluates were vacuum dried using a GeneVac EZ-2^plus^ (Genevac Ltd., Ipswich, United Kingdom).

### SDS-PAGE and Western Blotting

Total membrane protein extracts, FTs and eluates were analyzed by SDS-PAGE. Ten μg of proteins from each sample and 3 μL of BenchMark Protein Ladder (or 10 μL of BenchMark Prestained Protein Ladder for western blot, WB) were loaded on gels (Mini-PROTEAN TGX gels, 4–15%, BIO-RAD, United States). The dried eluates were suspended in 20 μL of 1x Laëmmli buffer and loaded totally on the gel. Proteins were stained with Coomassie Brilliant Blue R250 and gels were destained so as to obtain the optimum contrast.

The WB analyses were performed on PVDF membranes (transfer 1 h at 100 V in Tris-Glycine buffer inside a Mini Trans-Blot Electrophoretic Transfer cell) against adenylosuccinate lyase (ADSL C-11 at 1/500), α-adducin (C-4, 1/1000) and flotillin-2 (B-6, 1/6000) from Santa Cruz Biotechnology, United States.

#### First Antibody

Membranes were rinsed with PBST (1× PBS + 0.05% Tween-20) and blocked 1 h at RT under agitation with Top Block buffer (4% in PBST). Then the membranes were rinsed and washed 2 × 5 min in PBST. The blot was incubated overnight at 4°C under agitation with the primary antibody (ADSL at 1/500 in Top block buffer). After a quick rinse and three washes of 5 min at RT in PBST, the membranes were re-incubated in Top Block buffer 20 min at RT under agitation, and were incubated 1 h at RT under agitation with the secondary antibody (Polyclonal goat anti-mouse immunoglobulins HRP, Dako, Denmark) diluted at 1/10,000 in Top Block buffer. The membranes were finally washed several times in PBST and were incubated at least twice 30 min at RT under agitation. The ECL reaction was achieved using the ECL western blotting detection reagents (GE Healthcare, Little Chalfont, Buckinghamshire, United Kingdom) 1 min in the dark and the images were acquired by means of ImageQuant LAS 500 (GE healthcare, Uppsala, Sweden).

#### Membranes Stripping

After the ECL reaction, the blots were rinsed and washed 3 × 5 min in PBST and then stripped 40 min at RT under agitation in 20 mL of Restore^TM^ western blot stripping membrane (Thermo Scientific, Rockford, IL, United States). Then, the blots were rinsed and washed again 3 × 5 min in PBST before a new blocking 20 min at RT under agitation in Top Block buffer. Finally, they were rinsed, washed 2 × 5 min in PBST, and stored at 4°C for further immunodetection.

#### Second and Third Antibodies

After the membrane stripping, they were incubated with α-adducin diluted at 1/1,000 in Top Block buffer and the same procedure as before was applied. The last antibody tested was flotillin-2 diluted at 1/6,000 in Top Block buffer.

The WB membranes were finally stained with Ponceau red and scanned with a Personal Densitometer SI (GE Healthcare, United States).

#### Image Analyses and Statistic

Bands of interest were quantified by densitometry by means of the ImageQuant TL software (7.0, GE Healthcare, United States) and expressed as “volume.” The data were then corrected by the total protein loading detected on Ponceau red by densitometry (see Supplementary Material). The relative volumes were calculated as follows:

Relative VolumeProtein = VolumeProtein, ECL/VolumeWhole proteins, Ponceau

where Volume_Protein, ECL_ is the band volume of the protein of interest from WB and Volume_Whole proteins, Ponceau_ is the amount of loaded proteins determined by the densitometry analyses of whole lane from Ponceau red-stained membrane. Then, abundances were expressed as relative to short-stored ECs (effect of storage) or RBCs (RBCs vs. MVs) conditions.

*t*-test analyses were performed between long- and short-stored ECs using the software GraphPad Prism version 6.07 (GraphPad Software Inc.). Two-way ANOVA was used for flotillin-2 data and multiple comparisons. *p*-values lower than 0.05 were considered as significant.

### Proteomic Analysis

Each gel lane was cut in 8 bands (total membrane proteins and MVs experiments) and 8 or 13 bands (band 3 complexes in storage experiments) (**Supplementary Figure S4**), and proteins were in-gel digested (Delobel et al., 2012). Each excised band was treated and analyzed separately. Briefly, gel bands were washed with 50/50 ethanol/50 mM NH_4_HCO_3_. Proteins were reduced with 10 mM DTE in 50 mM NH_4_HCO_3_ 1 h at 37°C under agitation, and alkylated with 55 mM iodoacetamide in 50 mM NH_4_HCO_3_ 45 min at 37°C under agitation. Tryptic digestion was achieved by incubating gel bands in 12.5 ng/μL of trypsin (Sequencing Grade Modified Trypsin, Promega, Madison, WI, United States) in 50 mM NH_4_HCO_3_ and 10 mM CaCl_2_ overnight at 37°C. Peptides were extracted sequentially in 50/45/5 ethanol/H_2_O/FA then 70/25/5 ethanol/H_2_O/FA. Peptide extracts were vacuum dried and resuspended, after washing, in 20 μL of 78/20/2 H_2_O/FA/ACN for injection on LC coupled to mass spectrometry (LC-MS).

Ten microliter of peptide mixtures were analyzed onto an LC (UHPLC focused Thermo Scientific Dionex UltiMate 3000 Series from Thermo Scientific, Germering, Germany) coupled to an MS (amaZon ETD, Bruker Daltonik, Bremen, Germany) for protein identification. Peptide extract was loaded on a Dionex Acclaim PepMap RSLC column (300 μm × 15 cm, C18, 2 μm, 100 Å) at a 4 μL/min rate in a mixture of 95% eluent A (0.1% FA aqueous solution) and 5% of eluent B (H_2_O/ACN 20/80 + 0.8% FA). Peptides were separated through a 40 min gradient, rising to 25% of eluent B in 30 min, then to 40% in 5 min, and finally to 90% in 10 min. Mass spectra of separated peptides were acquired in the positive scan mode from 300 to 1500 *m*/*z*. An automatic MS/MS fragmentation was performed on the three most intense precursor ions of each spectrum. Precursor ions were excluded from MS/MS fragmentation after appearance in one spectrum, and released after 0.15 min. Precursors presenting a 5% increase in intensity during their exclusion time were reconsidered for MS/MS fragmentation.

Protein identifications were performed through ProteinScape (Version 3.0, Bruker Daltonics, Bremen, Germany) and based on Mascot Server (version 2.4, Matrix Science Ltd., Boston, MA, United States). Spectra were submitted to database search with the following parameters: Database = SwissProt; Taxonomy = Homo sapiens (human); Enzyme = Trypsin, allow up to 1 missed cleavage; Modifications = Carbamidomethyl on cysteine (fixed) and methionine oxidation (variable); Peptide tolerance = ±0.35 Da; MS/MS tolerance = ±0.35 Da; Peptide charge = 2+, 3+, and 4+; peptide score threshold = 15 (35 for one peptide only), accepted proteins if Mascot score > 40 (25 when re-analyzed under Mascot) and accepted peptides if Mascot score > 20.

Protein lists from biological replicates (*n* = 3 for effect of aging and *n* = 3 for RBCs vs. MVs) were merged together for comparisons (see Supplementary Material and **Supplementary Tables S3–S6** for details).

## Results

### Co-immunoprecipitation of Band 3 Complexes

**FIGURE 2:**
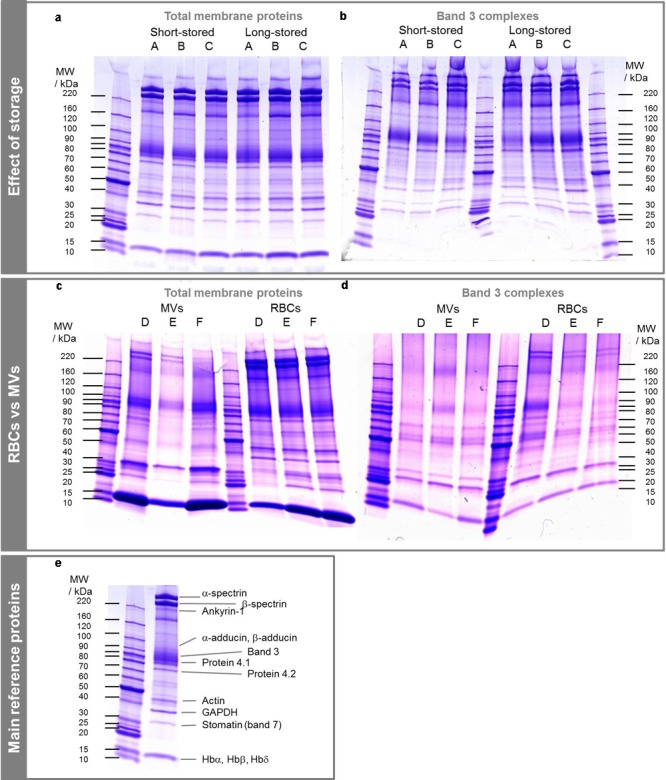
SDS-PAGE of total membrane proteins and band 3 complexes from RBCs and MVs. **(a,c)** Total membrane proteins. **(b,d)** Co-IP of total membrane proteins, i.e., band 3 complexes. Proteins were extracted with 1% DC buffer. **(e)** Location of main proteins on a total membrane protein extract. A–F stand for bag numbers. Band 3 dimers are observed around 220 kDa, especially in **(d)**.

Band 3 complexes were isolated from total membrane proteins from RBCs (**Figure 2a** and Supplementary Material). Membrane protein extracts showed a typical pattern including cytoskeleton proteins (e.g., α and β spectrins, protein 4.1), membrane proteins such as band 3 and proteins known to be localized to the membrane. FTs were similar to total membrane protein extracts because of the excess of incubated proteins regarding the binding capacity of the beads.

Six ECs were thus followed, total membrane proteins were extracted using DC-based buffer and band 3 complexes were isolated from RBCs at day 6 and day 42 (average), and from MVs using Co-IP. **Figure 2** shows the total membrane proteins (a and c) and protein contents after isolation of band 3 complexes (b and d). The protein distributions were quite similar between band 3 complexes and total membrane proteins. The main differences were the lower abundance of α and β spectrins relative to band 3, the lower abundance of low molecular weight proteins and the quasi absence of hemoglobin that was more important on bags D to F (IgG light chain was observed above 25 kDa). It emerges that hemoglobin derivatives do not bind to band 3 complexes but mainly to RBC membrane and that a large set of proteins is involved in these complexes, as expected. Protein 4.1 was less abundant in band 3 complexes than protein 4.2 even though their numbers of copies are equivalent (200,000 and 250,000 copies cell) (Burton and Bruce, 2011).

The MVs exhibit proteins mainly from the integral membrane, even though a few of them from the cytoskeleton, as the two spectrins and protein 4.1, and soluble proteins as hemoglobin (**Figure 2c**), were visible despite previous data (Lutz et al., 1977; Rubin et al., 2008; Salzer et al., 2008). As a consequence, the band 3 complexes isolated from MVs were free of spectrins which differs from RBCs-derived band 3 complexes (**Figure 2d**). The absence of proteins linking the skeleton to the membrane such as ankyrin, proteins 4.1 and 4.2 may suggest that mainly free complexes are expulsed through vesiculation.

### Proteomic Analysis of Membrane Extracts and Band 3 Complexes

In the view of characterizing the protein content and pointing out the possible effect of storage, proteins were identified by a bottom-up proteomic approach. The whole gel lanes were cut in several pieces (**Supplementary Figure S4**), proteins were in-gel digested and analyzed by LC-MS/MS, and analyses were merged together in order to obtain a list of proteins per lane (see **Supplementary Tables**).

#### RBC Membrane Proteins

Forty-five proteins were identified in RBC total membrane proteins. Sixteen belonged exclusively to total membrane proteins, 2 were only found in band 3 complexes and 27 were common to both total membrane proteins and band 3 complexes (see **Supplementary Table S1**). In particular, 34 proteins were identified in both short- and long-stored total membrane proteins, 5 only in long-stored and 4 others only in short-stored ECs extracts; whereas 19 and 29 proteins were identified in band 3 complexes stemming from short- and long-stored ECs, respectively. These lists are limited because of the type of instrument used but the goal was to identify potential differences. The majority of those are known proteins of RBC membranes (Pasini et al., 2010; D’Alessandro et al., 2017) and belong to the cytoskeleton: spectrins, actin, adducin; are glycoproteins: glycophorins A and C; and enzymes: GAPDH, aldolase, for instance.

#### Band 3 Complexes

Analyzing the effect of aging, it appears that only a few proteins co-immunoprecipitated with the band 3. In particular, three proteins were at least present in two long-stored bags and no more than one in short-stored bags (see **Table 1**). These latter proteins, namely adenylosuccinate lyase (ADSL), α-adducin and flotillin-2, were selected for WB analysis. Because of the MS instrument and the qualitative analysis, the selection of proteins was based on the presence or absence of proteins in samples and not on number of identified peptides or any other abundance scores.

**Table 1:** Proteomic differences between band 3 complexes isolated from long- and short-stored erythrocyte concentrate (ECs).

Protein name	Accession number	Long-stored ECs			Short-stored ECs		
		L-A	L-B	L-C	S-A	S-B	S-C
Adenylosuccinate lyase	P30566	–	+	+^∗^	–	–	–
Alpha-adducin	P35611	+	–	+	–	–	–
Flotillin-2	Q14254	+	+	+^∗^	–	+^∗^	–
*ATP-citrate synthase*	P53396	+	–	+^∗^	–	–	+
*Tropomodulin-1*	P28289	–	+	–	–	+^∗^	–
*Ubiquitin-conjugating enzyme E2N*	P61088	–	+	–	–	+^∗^	–
*Hemoglobin subunit delta*	P02042	–	+	–	–	–	–
*Phosphatidylinositol-5-phosphate 4-kinase*	P48426	+	–	–	–	–	–
*Ras-related protein Rap-1b*	P61224	–	–	+	–	–	–
*Ras-related protein Rap-2b*	P61225	–	–	+	–	–	–

*^∗^Additional identification using Mascot search engine 2.4.1 (July 2013).*

#### Microvesicles

Thirty-two proteins were found in common between RBC membrane proteins and vesicles, 26 specific to RBCs and 25 to MVs (see **Supplementary Table S2**). Of interest, acetylcholinesterase was enriched in MVs as reported by Salzer et al. (2002). As for the band 3 complexes, the numbers are lower with 11 in common, 5 specific to RBCs and 7 to MVs. Complement C4 and galectin-7 were found exclusively on MVs-derived band 3 complexes and they are associated to complement and pro-apoptotic activities, respectively.

### Quantification of ADSL, α-Adducin and Flotillin-2 by Western Blotting

**FIGURE 3:**
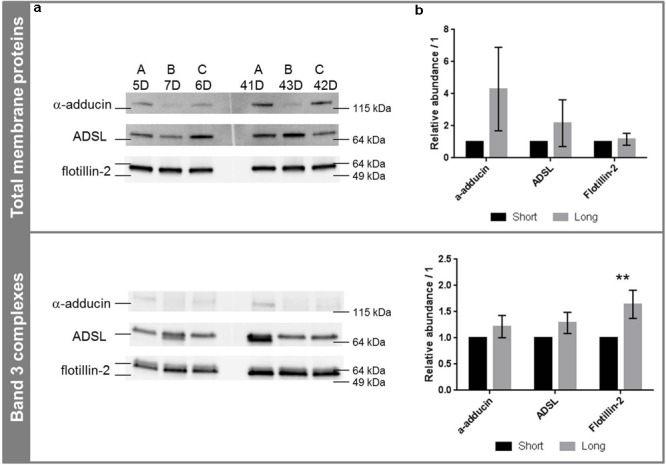
Western blotting analysis of α-adducin, ADSL and flotillin-2 in total membrane proteins and band 3 complexes. **(a)** Western blot images in total membrane proteins (top) and band 3 complexes (bottom) in function of storage. **(b)** Corresponding densitometric analyses expressed as the mean of the band volume (*n* = 3) and relative to the protein loading. ^∗∗^*p* < 0.01. Abundances were relative to short-stored ECs (see section “SDS-PAGE and Western Blotting” and Supplementary Material for details). A, B, and C stand for bag numbers; and D stands for days of storage.

Because of the sensitivity of WB, the segregation between the two groups was not as clear as reported by proteomics. The three proteins of interest were found in both short- and long-stored ECs (see **Figure 3**). No degradation was observed and the proteins were detected at their molecular weight (α-adducin is usually detected above the band 3, i.e., above 100 kDa). Their amounts tended to increase during storage without reaching statistical significances (**Figure 3b**). ADSL and α-adducin in band 3 complexes were preserved and tended to increase in the total membrane proteins; in particular α-adducin exhibited a fold change of 4.3 on membrane proteins extracted from long-stored EC (*p* = 0.088), specific to the membrane and not to band 3 complexes. The same tendency was observed in band 3 complexes, with marked effects for flotillin-2. It was clearly more abundant in long-stored ECs-derived band 3 complexes compared to short-stored ones with a fold change of 1.6 (*p* = 0.0024), but did not vary along storage in total membrane protein extracts (*p* = 0.45).

**FIGURE 4:**
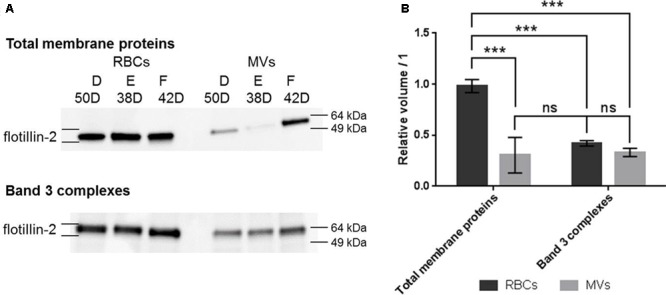
Western blotting analysis of flotillin-2 in total membrane proteins and band 3 complexes from RBCs and MVs. **(A)** Western blot images in total membrane proteins (top) and band 3 complexes (bottom) stemming from RBCs and MVs. **(B)** Densitometric analyses of flotillin-2. Relative volumes were expressed as the mean of the band volume (*n* = 3) and relative to the protein loading (see section “SDS-PAGE and Western Blotting” and Supplementary Material for details). D, E, and F stand for bag numbers; and D stands for days of storage. ^∗∗∗^*p* < 0.001.

The quantity of flotillin-2 was clearly less important in total membrane proteins extracted from MVs compared to RBCs (more than threefold decrease, *p* < 0.001) (**Figure 4B**). As for band 3 complexes, a non-significant 1.3-fold decrease was observed (*p* = 0.28). In addition, the levels of flotillin-2 in MVs were equivalent in total membrane proteins and band 3 complexes, which suggest that the excreted flotillin-2 was associated to band 3 complexes.

These results show an association of these proteins to band 3 complexes during the storage of RBCs. In particular, the amount of flotillin-2 was constant during the storage, it associated to band 3 complexes and it was released in MVs.

## Discussion

The number of band 3 partners is important (28) onto this major RBC membrane protein and corresponds to the proteins reported in the literature. These partners are peripheral and integral membrane proteins and have different functions such as the cytoskeleton organization, energy metabolism, transmembrane transport and blood group antigen. The band 3 complexes isolation showed good specificity as confirmed by Co-IP on naked beads (no protein adsorption) and isotypic control (weak adsorption of band 3 and other unidentified proteins, and release of Ab) (see Supplementary Material and **Supplementary Figure S2**), which enable to study the effect of storage. It has to be noticed that band 3 complexes were isolated by Co-IP without any differentiation of the types of complexes (i.e., ankyrin, junctional or free complexes). The buffer used allows to extract both membrane and cytoskeleton proteins, as already described (Delobel et al., 2012). Moreover, the use of DC in the extraction buffer may affect the association of a few proteins as protein 4.2. Indeed, washing of band 3 complexes-containing beads washed out protein 4.2 and spectrins (data not shown), as reported by Bruce et al. (2003).

The present study reveals the weak impact of *ex vivo* aging on band 3 complexes and 10 proteins were detected on long-stored EC-derived band 3 complexes. They play a role in metabolism activity, cytoskeleton organization and vesiculation such as Rap2 (Greco et al., 2006). A general accumulation trend of α-adducin on RBC membrane during the aging was observed, but it is not significant. α-adducin together with β subunit cap the fast-growing end of actin and recruit spectrins, and are involved in junction between band 3 and glucose transporter-1. It interacts also with dematin, a key protein in the organization of the junctional complex. It was reported (in mouse) that its absence induces the loss of adducin, actin and spectrin, and changes the RBC morphology (Lu et al., 2016). But the present data do not allow to conclude on this key protein (dematin was found both in short- and long-stored RBCs but was absent from MVs, see **Supplementary Tables S1, S2**).

The differences were significantly confirmed by WB for flotillin-2 that associates to band 3 complexes during the storage even though the data do not allow to specifically identify the proteins or the lipids driving the association. Flotillin-2 is a lipid raft-associated protein (Salzer and Prohaska, 2001) that is less abundant in MVs than in RBCs as previously reported (Salzer et al., 2002, 2008; Kriebardis et al., 2008). Since flotillin-2 was mainly found in MVs associated to band 3 complexes (**Figure 4**), it is speculated that flotillin-2 [known to be tightly associated with the cytoskeleton (Ciana et al., 2005)] could be specifically eliminated through the microvesiculation process at the end of the storage. In addition, stomatin, known to be enriched in MVs (Salzer et al., 2008), was also reported to be linked to flotillins and found in high molecular weight complexes (Rungaldier et al., 2013). These interactions may play a role in this process but the mechanism is unknown. This lipid raft-based process was also observed in calcium-induced microvesiculation (Salzer et al., 2002) though involving different sets of proteins (Prudent et al., 2015a). These observations are in agreement with the theory developed by Gov et al. (2009) where mobile components and breakage of the band 3-ankyrin anchors lead to vesiculation and morphology changes (Satchwell et al., 2016). Of course, other known mechanisms may be involved such as the loss of ATP-dependent aminophospholipid translocase (Salzer et al., 2008), the exposure of phosphatidyl serine or the accumulation of oxidized proteins (Kriebardis et al., 2008; Gov et al., 2009).

## Conclusion

The isolation of band 3 complexes from RBCs revealed the high number of protein partners involving several functions. Moreover, it points out and confirms the association of flotillin-2 to band 3 complexes during the storage of RBCs and in storage-induced MVs. The role played in MVs remains unclear and the isolation of different types of complexes will be required to identify if a specific complex is involved in the binding of this protein during the storage. The stability of the protein complexes is influenced by protein-protein interactions and cell membrane organization. Post-translational modifications such as protein oxidation (Delobel et al., 2016; Reisz et al., 2016) or phosphorylation (Husain-Chishti et al., 1988; Harrison et al., 1991) impact the organization of the complexes. Therefore, these protein modifications could be further investigated during RBCs aging to provide more information on the storage lesions.

## Author Contributions

MP developed the co-IP, followed the EC bags, ran the proteomics, analyzed the data, and wrote the article. CB made the co-IP for the aging of ECs and in-gel digested the proteins. AH carried out the work on MVs. JD, NL, and J-DT reviewed the data and the manuscript. All authors read and approved the final version of the paper.

## Conflict of Interest Statement

The authors declare that the research was conducted in the absence of any commercial or financial relationships that could be construed as a potential conflict of interest.

## Supplementary Material

FIGURE S1 Band 3 complexes captured using different antibodies against band 3. BRIC 6 (internal epitope), 155 (C-terminal) and 170 (N-terminal). NuPAGE Novex 4–12% gel from Invitrogen. Ten μg of proteins loaded and 5 μg of antibodies. FT, flow through (identical to protein extract); E, eluate; Ab, antibodies. BRIC 6 and 170 were chosen for co-IP.

FIGURE S2 RBC total membrane proteins, adsorption on naked M-270 Epoxy beads and isotypic control. **Left**: NuPAGE Novex 4–12% gel from Invitrogen. Ten μg of proteins loaded. FT, flow through (identical to protein extract); E, eluate samples from co-IP on naked beads. M: molecular weight marker. **Right**: SDS-PAGE 4–15% gel from Bio-Rad. Ten μg of proteins loaded. E: eluate samples from co-IP on naked beads (1), on beads coated with an IgG against C23 (IgG, D-6, Santa-Cruz) (2), and on beads coated with BRIC 6 and BRIC 170 against band 3. No adsorption was observed on naked beads and only a few proteins were observed on the isotypic control (see text for details).

FIGURE S3 SDS-PAGE analysis of co-IP band 3 complexes. In order to test the specificity of the beads and antibodies used, band 3 complexes were denatured with 1% SDS (+ or -) as is: before co-IP protein extract was incubated 5 min at RT under agitation in DC buffer containing 1% SDS. Red bars point to main differences. Green arrows are for the presence of released antibodies. FT, flow through (identical to protein extract); E, eluate samples. “+” and “-” signs stand for the presence or absence of SDS in the sample, respectively.

FIGURE S4 Band excision of band 3 complexes for proteomics. **Top**: Effect of storage (bags A, B, and C). For bag B, additional bands were included as shown on the bottom right for numbering. **Bottom**: RBCs vs MVs (bags D, E, and F). Each red box represents a digested band. The same mapping was used for the total membrane proteins.

FIGURE S5 Western blotting analyses of α-adducin, ADSL and flotillin-2 in total membrane extracts **(left)** and band 3 complexes **(right)** in function of RBC storage. Raw images. Acquisition times of 3 min for ADSL and flotillin-2, and 5 min for α-adducin. Green arrow: detection of released antibodies; blue arrow: remaining ADSL due to sequential detection. A, B, and C stand for the bags and D is for days of storage. Colored bands are for the MW markers (Pink band: 64 kDa).

FIGURE S6 Western blotting analyses of flotillin-2 in total membrane extracts **(left)** and band 3 complexes **(right)** in function of their origin (RBCs or MVs). Raw images. Acquisition time of 3 min. Green arrow: detection of released antibodies. D, E, and F stand for the bags and D is for days of storage. Colored bands are for the MW markers (Pink band: 64 kDa).

FIGURE S7 Example of image analyses by densitometry. **Left**: Western blotting of flotillin-2 on total membrane proteins. **Right**: Corresponding Ponceau red-stained membrane showing the total protein contents.

FIGURE S8 Levels of protein expression in function of aging. Densitometric analyses of α-adducin, ADSL and flotillin-2 in total membrane proteins **(left)** and band 3 complexes **(right)**. Relative volumes were expressed as the mean of the band volume (*n* = 3) and relative to the protein loading. They were obtained for each protein and cannot be compared to another one (see section “SDS-PAGE and Western Blotting” and Supplementary Material for details). ^∗∗^*p* < 0.01.

FIGURE S9 Levels of flotillin-2 expression in RBCs and MVs. Densitometric analyses of flotillin-2 in total membrane proteins and band 3 complexes in function of the origin (RBCs or MVs). Relative volumes were expressed as the mean of the band volume (*n* = 3) and relative to the protein loading. Abundances were relative to RBCs (see “SDS-PAGE and Western Blotting” and Supplementary Material for details). ^∗∗∗^*p <* 0.001; ns, non-significant.

TABLE S1 Proteomic data. Effect of storage.

TABLE S2 Proteomic data. RBCs vs. MVs.

TABLE S3 Detailed proteomic data for **Figure 2a**.

TABLE S4 Detailed proteomic data for **Figure 2b**.

TABLE S5 Detailed proteomic data for **Figure 2c**.

TABLE S6 Detailed proteomic data for **Figure 2d**.

## Abbreviations

ACN: acetonitrile
Co-IP: co-immunoprecipitation
DC: deoxycholate
EC: erythrocyte concentrate
FA: formic acid
FT: flow through
LC: liquid chromatography
MS: mass spectrometry
MV: microvesicle
PBST: phosphate buffered saline tween
RBC: red blood cell
RT: room temperature
SAGM: saline-adenine-glucose-mannitol
