# Supplementary material for: Proteomics of Stored Red Blood Cell Membrane and Storage-Induced Microvesicles Reveals the Association of Flotillin-2 With Band 3 Complexes
Source: Front Physiol. 2018 May 4;9:421. doi: 10.3389/fphys.2018.00421 (PMC5945891; doi:10.3389/fphys.2018.00421)
Supplement: FIGURE S1 — Band 3 complexes captured using different antibodies against band 3. BRIC 6 (internal epitope), 155 (C-terminal) and 170 (N-terminal). NuPAGE Novex 4–12% gel from Invitrogen. Ten μg of proteins loaded and 5 μg of antibodies. FT, flow through (identical to protein extract); E, eluate; Ab, antibodies. BRIC 6 and 170 were chosen for co-IP. [file Data_Sheet_1.docx]

Supplementary Material

**Proteomics of stored red blood cells membrane and storage-induced microvesicles reveals the association of flotillin-2 with band 3 complexes**

Michel Prudent^1,2^*, Julien Delobel^1^, Aurélie Hübner^1^, Corinne Benay^1^, Niels Lion^1,2^ and Jean-Daniel Tissot^1,2^

*** Correspondence:** [michel.prudent@itransfusion.ch](mailto:michel.prudent@itransfusion.ch)

# Co-IP

Four times 6 mL of ECs were collected from one EC after 8 days (8D) and 42 days (42D) of storage for the development of the Co-IP assay. RBCs were washed twice in 0.9% NaCl (2 v) and spun down at 2000g during 10 min at 4°C. RBCs were lyzed by incubation 1 h at 4°C in a hypotonic 0.1x PBS solution under agitation (2 v 0.1x PBS for 1 v of cells).

Membranes (from test bag) were separated by centrifugation at 11’000g during 30 min at 4°C. Membranes were thus washed 7x with 0.1x PBS in 15-mL tubes, transferred to 1.5-mL tubes and washed 5 more times for 42-day-old membranes and 1 more time for 8-day-old membranes (centrifugation at 18’620g, 30 min, 4°C).

## Antibodies against band 3

Different antibodies and quantities were tested for optimizing the yield of band 3 complex captured (data not shown). The optimal co-IP was obtained by using a combination of two antibodies (BRIC 6 and 170 specific to internal and N-ter band 3 sequence, respectively, Supplementary Figure 1) for a total of 105 μg covalently bound to 7.5 mg of magnetic beads (representative band 3 complexes pattern is shown in Supplementary Figure 1). One hour of incubation at 4°C proved to be enough to immunoprecipitate these complexes.

**
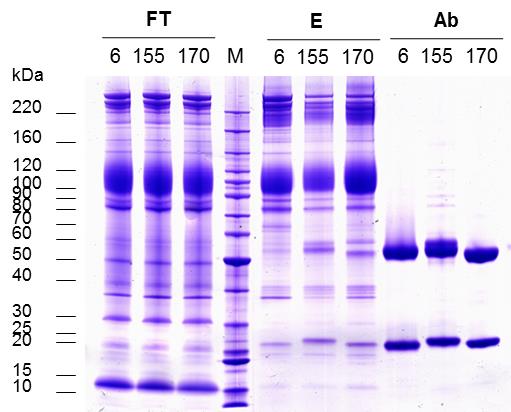
**

**Supplementary Figure S1: Band 3 complexes captured using different antibodies against band 3.** BRIC 6 (internal epitope), 155 (C-termimal) and 170 (N-terminal). NuPAGE Novex 4-12% gel from Invitrogen. Ten μg of proteins loaded and 5 μg of antibodies. FT: flow through (identical to protein extract), E: eluate, Ab: antibodies. BRIC 6 and 170 were chosen for co-IP.

## Adsorption on naked beads and isotypic control

Naked beads did not show any protein bindings (Supplementary Figure 2) when incubated with total membrane proteins (traces of spectrins were slightly observed). As for the isotypic control a dark track was observed and a few proteins were adsorbed on the IgG-coated beads: mainly IgG released from the beads, a band at the MW of band 3 without the smear effect and a few other proteins. No specific adsorption of band 3 complexes was observed compared to eluates. Moreover, the beads used were equivalent for all the experiments (beads obtained from pool-and-split after coating with the anti-band 3) and these few adsorptions were considered as negligible.

## Complex denaturation

Complexes were denatured with 1% SDS after extraction under native conditions from membranes. The eluate resulting from such co-IP is shown in Supplementary Figure 3 (lane 8). Even though the pattern looks similar to control (co-IP in native conditions, lane 6) because of the band 3, several differences are clear. First of all, the band 3 and band 3 dimers around 200-220 kDa are more intense compared to the whole proteins within the lane. Since band 3 is sensitive to sample preparation, boiling of samples was strictly limited to 5 min. Second, several proteins were absent within the high and low molecular weight regions, as highlighted by red bars. Finally, the spectrins (α and β) that were still eluated, were probably tightly linked to band 3 since naked beads and isotypic experiments showed poor non-specific interactions (Supplementary Figure 2). Other bands belong to the antibodies used for co-IP (BRIC 6 and BRIC 170) which slightly eluate with the band 3 complexes (Supplementary Figure 1).


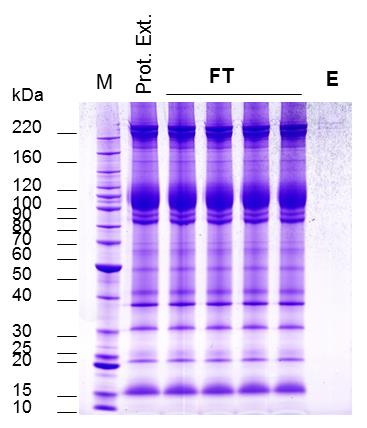

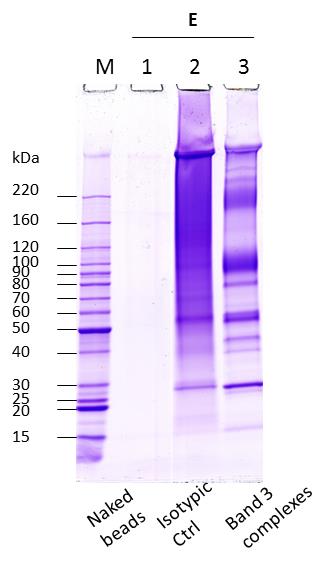


**Supplementary Figure S2: RBC total membrane proteins, adsorption on naked M-270 Epoxy beads and isotypic control.** Left: NuPAGE Novex 4-12% gel from Invitrogen. Ten μg of proteins loaded. FT: flow through (identical to protein extract), E: eluate samples from co-IP on naked beads. M: molecular weight marker. Rigth: SDS-PAGE 4-15% gel from Bio-Rad. Ten μg of proteins loaded. E: eluate samples from co-IP on naked beads (1), on beads coated with an IgG against C23 (IgG, D-6, Santa-Cruz) (2), and on beads coated with BRIC 6 and BRIC 170 against band 3. No adsorption was observed on naked beads and only a few proteins were observed on the isotypic control (see text for details).


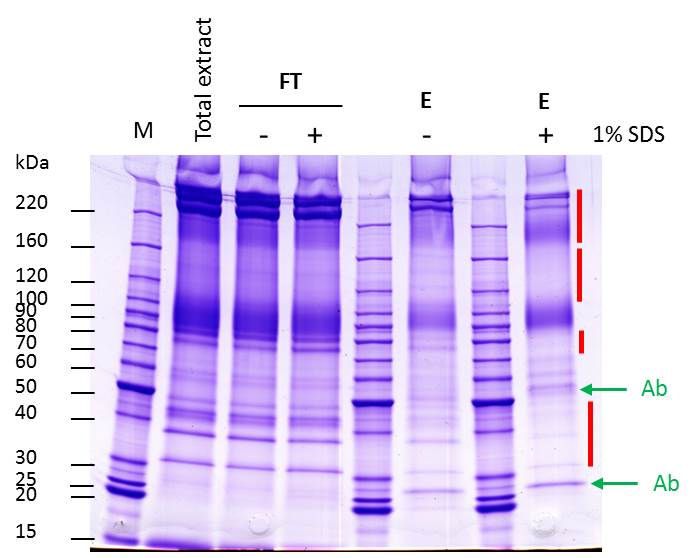


**Supplementary Figure S3:** **SDS-PAGE analysis of co-IP band 3 complexes.** In order to test the specificity of the beads and antibodies used, band 3 complexes were denatured with 1% SDS (+ or -) as is: before co-IP protein extract was incubated 5 min at RT under agitation in DC buffer containing 1% SDS. Red bars point to main differences. Green arrows are for the presence of released antibodies. FT: flow through (identical to protein extract), E: eluate samples. “+” and “-“ signs stand for the presence or absence of SDS in the sample, respectively.

# Proteomics

Each gel lane was cut in 8 bands (total membrane proteins and MVs experiments) and 8 or 13 bands (band 3 complexes in storage experiments), and proteins were in-gel digested (Delobel et al., 2012). Gels for band 3 complexes (storage and MVs experiments) are shown in Supplementary Figure 4 (excision maps for total membrane extracts were not shown but were similar).


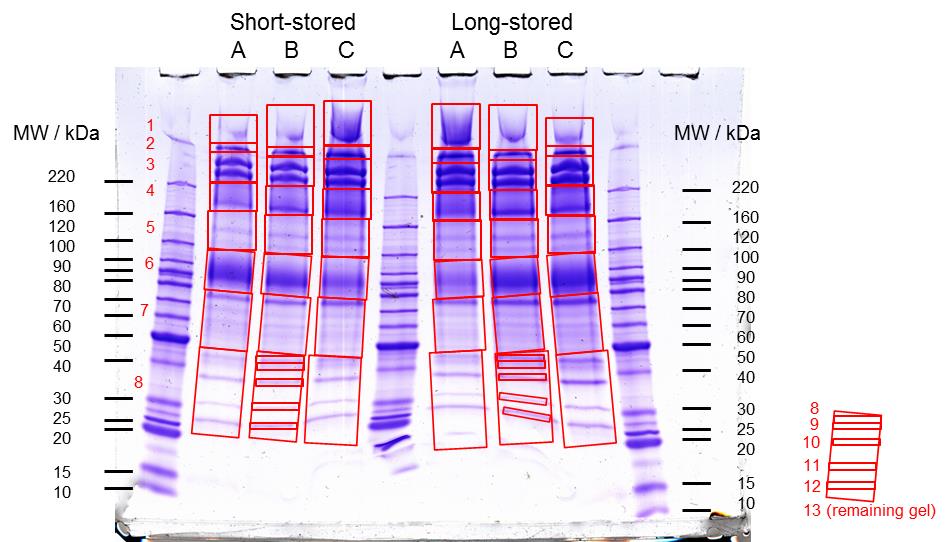


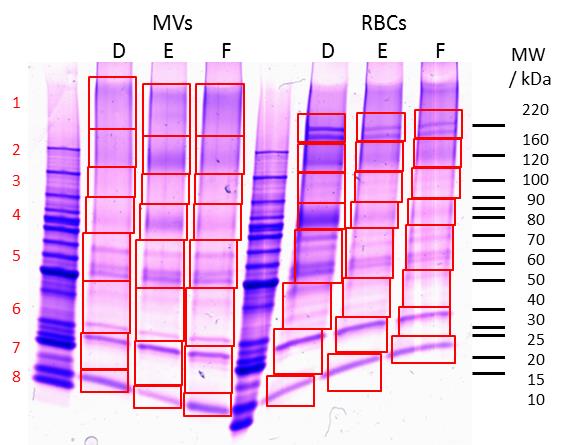


**Supplementary Figure S4:** **Band excision of band 3 complexes for proteomics**. Top: effect of storage (bags A , B and C). For bag B, additional bands were included as shown on the bottom right for numbering. Bottom: RBCs vs MVs (bags D, E and F). Each red box represents a digested band. The same mapping was used for the total membrane proteins.

Ten microliter of extracted peptide mixtures were analyzed onto an LC (UHPLC focused Thermo Scientific Dionex UltiMate 3000 Series from Thermo Scientific, Germering, Germany) coupled to an MS (amaZon ETD, Bruker Daltonik, Bremen, Germany) for protein identification.

Protein identifications were done through ProteinScape (version 3.0, Bruker daltonics, Bremem, Germany) and based on Mascot Server (version 2.4, Matrix Science Ltd, Boston, MA, USA). Protein lists from each band were merged together in order to obtain a protein list per lane (lanes A to F) and per condition (aging or RBCs vs MVs). Then, protein lists from biological replicates were merged together for comparisons (short- vs long-stored or RBCs vs MVs). Detailed data per lane are shown in Supplementary Tables 3 to 6).

# Co-immunoprecipitation – Western blot


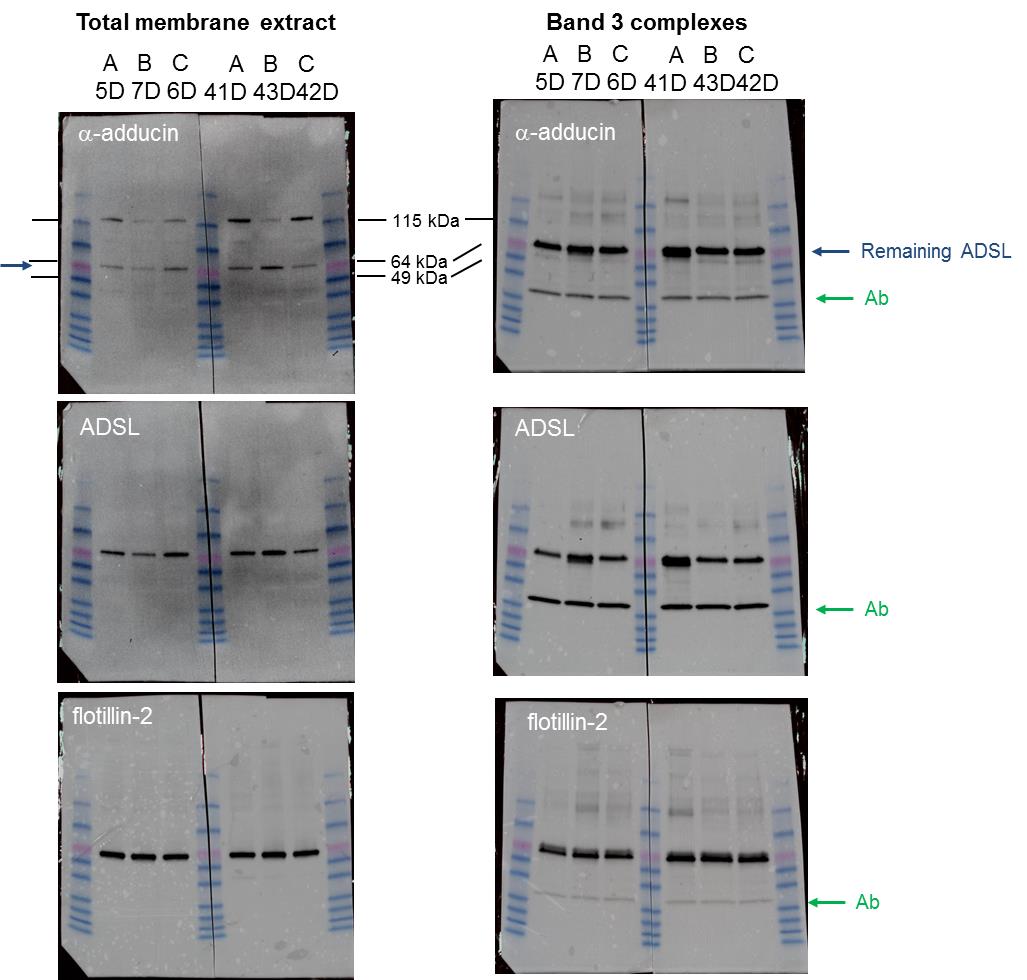


**Supplementary Figure S5:** **Western blotting analyses of α-adducin, ADSL and flotillin-2 in total membrane extracts (left) and band 3 complexes (right) in function of RBC storage.** Raw images. Acquisition times of 3 min for ADSL and flotillin-2, and 5 min for α-adducin. Green arrow: detection of released antibodies; blue arrow: remaining ADSL due to sequential detection. A, B and C stand for the bags and D is for days of storage. Colored bands are for the MW markers (Pink band: 64 kDa).


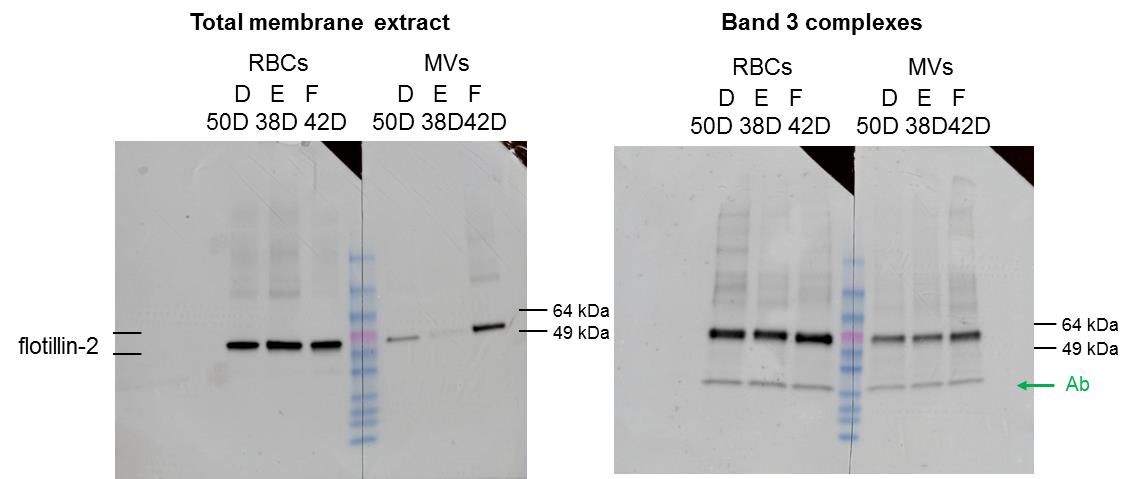


**Supplementary Figure S6:** **Western blotting analyses of flotillin-2 in total membrane extracts (left) and band 3 complexes (right) in function of their origin (RBCs or MVs).** Raw images. Acquisition time of 3 min. Green arrow: detection of released antibodies. D, E and F stand for the bags and D is for days of storage. Colored bands are for the MW markers (Pink band: 64 kDa).

# Quantification of protein level

Supplementary Figure 7 shows an example of images used for the quantification of flotillin-2. The volumes (arbitrary unit) of flotillin-2 were measured by densitometry (left). Then, the total protein amounts loaded on the gel were quantified on the whole lane on a Ponceau-stained membrane (right). The protein levels were expressed as “Relative volume” and were obtained as follows:

$${Relative Volume}_{Protein}={{Volume}_{Protein,ECL}}/{{Volume}_{Whole proteins, Ponceau}}$$

where Volume_Protein, ECL_ is the band volume of the protein of interest from western blot and Volume_Whole protein, Ponceau_ is the amount of loaded proteins determined by the densitometry analyses of whole lane from Ponceau red-stained membrane. Therefore, values higher or lower than 1 do not stand for any biological meaning from protein to protein. In addition, abundances were expressed as relative to short-stored ECs (effect of storage) or RBCs (RBCs vs MVs) conditions.

The same method was applied to all the images (data from bags A to F).


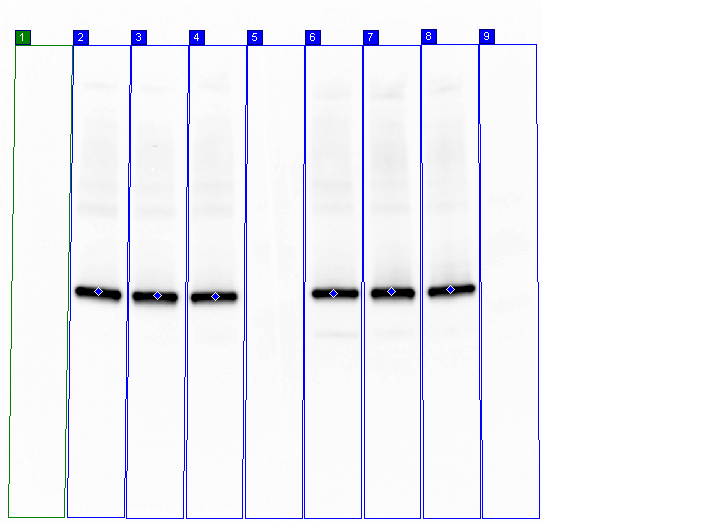

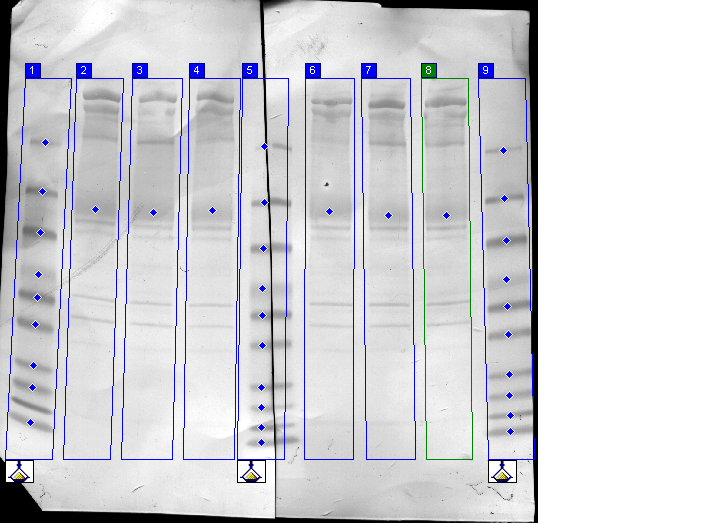


**Supplementary Figure S7: Example of image analyses by densitometry.** Left: western blotting of flotillin-2 on total membrane proteins. Right: corresponding Ponceau red-stained membrane showing the total protein contents.


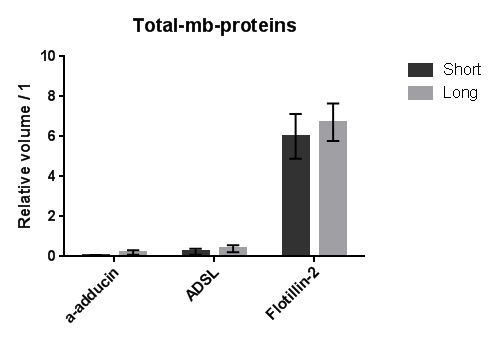

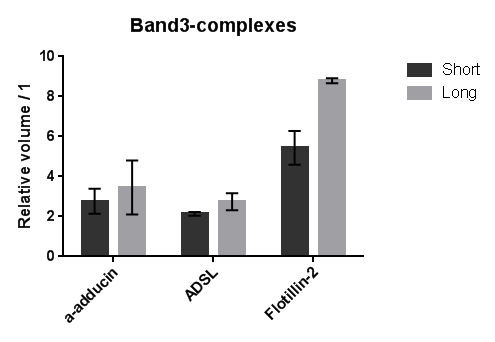


**

*

**Supplementary Figure S8:** **Levels of protein expression** **in function of aging.** Densitometric analyses of α-adducin, ADSL and flotillin-2 in total membrane proteins (left) and band 3 complexes (right). Relative volumes were expressed as the mean of the band volume (*n* = 3) and relative to the protein loading. They were obtained for each protein and cannot be compared to another one (see section 2.6 and Supplementary material for details). ** p < 0.01.


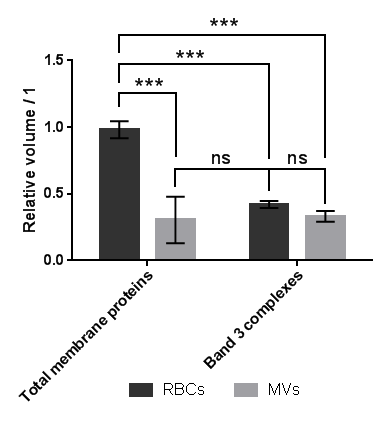

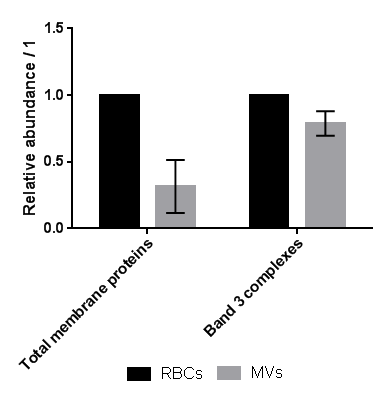


***

**Supplementary Figure S9:** **Levels of flotillin-2 expression in RBCs and MVs.** Densitometric analyses of flotillin-2 in total membrane proteins and band 3 complexes in function of the origin (RBCs or MVs). Relative volumes were expressed as the mean of the band volume (*n* = 3) and relative to the protein loading. Abundances were relative to RBCs (see section 2.6 and Supplementary material for details). *** *p* < 0.001, ns: non-significant.

# References

Delobel, J., Prudent, M., Rubin, O., Crettaz, D., Tissot, J.-D., and Lion, N. (2012). Subcellular fractionation of stored red blood cells reveals a compartment-based protein carbonylation evolution. *Journal of Proteomics* 76**,** 181-193. doi: 10.1016/j.jprot.2012.05.004.
